# Country ownership and capacity building: the next buzzwords in health systems strengthening or a truly new approach to development?

**DOI:** 10.1186/1471-2458-12-531

**Published:** 2012-07-20

**Authors:** Jessica Goldberg, Malcolm Bryant

**Affiliations:** 1 96 Cummings Avenue, 1-L, Quincy, MA, 02170, USA; 2 Boston University Center for Global Health and Development, 801 Massachusetts Avenue, Boston, MA, 02118, USA

**Keywords:** Country ownership, Capacity building, Organizational development, Global health initiative, Paris declaration on aid effectiveness

## Abstract

**Background:**

During the last decade, donor governments and international agencies have increasingly emphasized the importance of building the capacity of indigenous health care organizations as part of strengthening health systems and ensuring sustainability. In 2009, the U.S. Global Health Initiative made country ownership and capacity building keystones of U.S. health development assistance, and yet there is still a lack of consensus on how to define either of these terms, or how to implement “country owned capacity building”.

**Discussion:**

Concepts around capacity building have been well developed in the for-profit business sector, but remain less well defined in the non-profit and social sectors in low and middle-income countries. Historically, capacity building in developing countries has been externally driven, related to project implementation, and often resulted in disempowerment of local organizations rather than local ownership. Despite the expenditure of millions of dollars, there is no consensus on how to conduct capacity building, nor have there been rigorous evaluations of capacity building efforts. To shift to a new paradigm of country owned capacity building, donor assistance needs to be inclusive in the planning process and create true partnerships to conduct organizational assessments, analyze challenges to organizational success, prioritize addressing challenges, and implement appropriate activities to build new capacity in overcoming challenges. Before further investments are made, a solid evidence base should be established concerning what works and what doesn’t work to build capacity.

**Summary:**

Country-owned capacity building is a relatively new concept that requires further theoretical exploration. Documents such as The Paris Declaration on Aid Effectiveness detail the principles of country ownership to which partner and donor countries should commit, but do not identify the specific mechanisms to carry out these principles. More evidence as to how country-owned capacity building plays out in practice is needed to guide future interventions. The Global Health Initiative funding that is currently underway is an opportunity to collect evaluative data and establish a centralized and comprehensive evidence base that could be made available to guide future country-owned capacity building efforts.

## Background

In 2009, the United States initiated a dramatic policy shift regarding the provision of foreign assistance with the introduction of the Global Health Initiative (GHI). The initiative aims to invest up to $63 billion dollars over six years to help partner countries improve health outcomes through the strengthening of health systems, and seeks to enable U.S. governmental agencies providing foreign assistance to become more efficient, accountable, and effective in their efforts to “help countries lift themselves out of poverty” [1]. The underlying principles of the GHI involve building country-level capacity to manage and operate health programs and to encourage country-ownership of health efforts through investing in country-led plans [2]. This effort by the United States is simply the latest in a series of efforts targeted on country ownership coming through documents such as the Paris Declaration on Aid Effectiveness of 2005, the Accra Agenda for Action of 2008, and guidelines from the Global Fund to Fight AIDS, Tuberculosis, and Malaria.

The intersection of capacity building and country ownership merits further theoretical consideration. Capacity building is an approach to strengthening organizations that is common to a variety of different sectors, including business and health program management. It is a term that has been used with such frequency and variety of interpretation that its true meaning has become obscured. Capacity building in foreign assistance efforts has in the past often entailed donor country organizations driving the design and implementation of their technical assistance programs without equal participation from partner country organizations. The GHI approach to foreign assistance explicitly breaks with past behavior and promotes local involvement by embracing the notion of “country-ownership”. This is a capacity building strategy that shifts the leadership of and responsibility for health promotion efforts onto the partner country and its organizations on the ground. If the GHI’s emphasis on promoting country ownership in capacity building is carried out in practice, a huge influx of development funding will soon be allocated to a new concept, “country-owned capacity building”, that is not clearly understood by practitioners or researchers alike.

This paper examines the relationship between organizational capacity building and the concept of country ownership and proposes a working definition of country-owned capacity building. A simple conceptual framework for implementing country-owned capacity building efforts will also be described in order to support partner/donor organization collaboration in capacity building efforts. The intended audiences of this paper are the partner organizations that are the focus of capacity building efforts, and the donor organizations that play a variety of direct or indirect roles in capacity building initiatives (e.g. providing funding, engaging in implementation activities, etc..). This working definition and framework will enable these organizations to develop a clearer understanding of the distinctive characteristics and goals of country-owned capacity building. Emerging lessons learned in promoting successful country-owned capacity building will be identified and analyzed to provide a reference to organizations involved in similar efforts.

## Discussion

### Defining capacity building

There is a general consensus in the literature that capacity building is a difficult concept to define [3-5]. Some explanations for this difficulty include the expansiveness of the term, its increasing popularity since the 1990s, and the inconsistency with which it has been applied [3,6,7]. These factors work in concert to hinder analytical understanding of the concept’s meaning. It is, however, possible to identify commonalities and differences among the various capacity building definitions in circulation.

Capacity building is a common concept across health and business sectors and is often described as either a process or an action aimed at the development of one or more organizational components [4,8-11]. It is also defined as a means to improving an organization’s ability to meet its stated goals [8,12], achieve its mission or enhance its efficiency or effectiveness [10,13-15]. Other commonalities among capacity building definitions include that these efforts are generally linked in some way to improved performance of an organization and related to the core tasks and functions of an organization [8,16,17].

Despite the commonalities in definitions, capacity building is generally described more precisely within the business and management literature than in the health literature. Examples include terms such as organizational innovation, organizational development, absorptive capacity; strategic management and strategic transformation are described as approaches to building organizational competitiveness, effectiveness, growth, sustainability and responsiveness [18-21]. The differences in terminology may be reflective of differences in the organizational objectives in each sector [22]. In for-profit business, organizational expansion and increased profit margins are often the desired outcomes of capacity building efforts [23]. In the non-profit/non-governmental organization sector, organizations achieve success by fulfilling their missions often through service provision; capacity building in this context is directed toward enhancing programming and service delivery [24].

### Concepts informing the definition of country-owned capacity building

In order to establish a working definition of capacity building, it is necessary to examine and incorporate additional concepts. Capacity building is essentially an internal process that can be enhanced or accelerated by outside groups [16,25]. In resource-poor settings, there may be limited organizational energy (time, skills, expertise, funds, facilities and equipment) to direct toward activities such as strategic planning and needs assessments, which should ideally precede capacity building activities [22]. Collaborations between donor/partner organizations can maximize the effectiveness of capacity building ventures by focusing on key activities [26]. Unfortunately, capacity building efforts driven by outside organizations or funders are often ineffective in cultivating a sense of ownership within the partner organization [27].

All organizations must already have some level of capacity as a pre-condition to effectively build more capacity [28]. Organizational readiness for capacity building is comprised of qualities such as the organization’s willingness to question itself, its understanding of its own mission and goals, and the level of commitment from key organizational stakeholders to participate in capacity building efforts [7]. The development of new organizational capacities is often reflected by an organization’s openness to change and the ability to adapt to shifts in its environment. Successful change efforts are largely predicated on the willingness of the organization’s leadership to support and facilitate that process [7,23,29,30]. At the same time, individuals are the building blocks of organizational capacity and the willingness of individuals and groups within the organization to change their behaviors can significantly affect the success of a capacity building intervention [29,31,32].

If capacity building is to become a process that is not externally driven, but is locally owned, it is important to reflect on some of the lessons learned over the last three decades. Firstly, technical assistance on the part of donor organizations is must be carefully tailored, given the wide variety of needs and resources present among partner organizations [33]. Secondly, it should not be assumed that past successful capacity building initiatives could be replicated exactly under different conditions. Thirdly, capacity building should be a participatory process, both as a collaborative venture between the partner and donor organization and also in terms of engaging staff and stakeholders at all levels of the partner organization [27,34]. Fourthly, it is necessary to identify relevant indicators to monitor and evaluate the success of a given capacity building effort. Fifthly, capacity building is a continuous process wherein the organizations involved set and monitor time-limited goals but are constantly and actively aspiring to improved levels of functioning [12,31,35]. Finally, capacity building should stress building an organization’s ability to adapt and respond in changing environments [4].

### Capacity building and country-ownership

Country ownership is an approach to development assistance that strives to empower partner countries to take, "effective leadership over their development policies, and strategies and co‒ordinate development actions" [36]. The Paris Declaration for Aid Effectiveness of 2005 and the Accra Agenda for Action of 2008 detail the principles of country ownership, which involve the partner country defining its own development priorities and designing and leading programs that promote these priorities. The country ownership paradigm represents a departure from past international development approaches, which were largely externally driven. The capacity building theory and tools prior to the advent of country-ownership were generally targeted for use by external organizations. Country-owned efforts involve building-up in-country technical knowledge and promoting organizational sustainability to minimize partner countries’ reliance on expensive, external expertise that is necessarily time-limited [36].

Country-ownership recognizes the need for endogenous drivers toward development in partner countries [37]. It is rooted in ideological values, including self-determination, and advanced by evidence-based analysis of effective approaches [37]. Country-ownership requires partner countries to accept full responsibility for success of the development activities. The assumption states that a partner country will be more likely to allot the necessary resources to ensure the full implementation of a program if it is accountable for its outcomes [38]. Critics of the concept cite theoretical difficulties in achieving country-ownership given the heterogeneity of interests among key stakeholders in the partner country, and challenges to designing a representative consultative process in planning and implementing programs [39].

The focus of country-owned capacity building should be to give organizations skills necessary to respond to challenges, solve problems and build capacity independently in the future. The Global Fund experience in building country-ownership aims to encourage partner country organizations to generate technically sound proposals for funding that are reflective of country needs, and to ensure that nongovernmental organizations play an instrumental role in designing, implementing and overseeing programs [40]. Successful country-owned capacity building projects echo the importance of inclusiveness in the planning process and excellent working relationships between partner/donor organizations that produce true partnerships [41,42]. Donor organizations require expertise in building governance, management systems and organizational development in order to effectively build capacity in collaboration with partner organizations [22,43].

### Introducing a working definition of capacity building in the context of country ownership

Having now explored the terms “capacity-building” and “country-ownership” in closer detail, it is possible to offer a working definition that combines the two concepts. This definition aims to clarify the interaction between the terms and to account for specific considerations that must be addressed by organizations engaging in country-owned capacity building efforts. Capacity building in the context of country ownership is therefore, “a continuous and participatory process undertaken independently or in collaboration with external partners to empower the organization to systematically identify and respond to its institutional needs and the needs of the population it serves in order to better meet its stated mission and goals, solve problems, implement change and increase efficiency”.

### The use of capacity building frameworks

A theoretical framework is a tool that can help to operationalize country-owned capacity building by providing a structured way in which to carry out the processes described in the definition above. There are many examples of capacity building frameworks, both in the peer reviewed and gray literature [22,44-47]. These frameworks share a number of commonalities, including the identification of organizational “core competencies” that each require a certain level of capacity in order for an organization to be effective.

Organizational effectiveness is contingent upon the interplay of these core competencies, examples of which include governance, financial management, leadership, technical capacities, human resources and systems and infrastructure. Core competencies are also called ‘domains’ or ‘elements’ of organizational efficiency, and can be described by a variety of individual terms (i.e. ‘aspirations’ versus ‘mission and goals’). Each core competency houses a number of smaller capacities, sub-domains, or “target areas of intervention”, that are implicit within the larger competency (i.e. financial management includes budget preparation, internal auditing, etc.). The relative importance of each core competency remains unclear, as does their respective temporal ordering, which precludes agreement among practitioners about which capacity building framework is superior [47].

Frameworks also highlight the steps in completing capacity building projects. Often, these steps include an initial assessment phase wherein the organization identifies areas of needed development [22,44,46,48,49]. A period of planning and implementing capacity building initiatives generally follows the assessment stage and monitoring and evaluating the initiative is often the final stage. These stages are broad and general and can be deconstructed further into to their component parts in order to allow for greater understanding. Table 1 provides a description of the steps in a capacity building framework for county-owned projects.

**Table 1:** Steps in country-owned capacity building framework

**Step**	**Action**
1.	Conduct an organizational self-assessment to find areas of strength and weakness
	i) Define the impact of identified weaknesses on performance
	ii) Prioritize weaknesses for intervention
	iii) Break down weaknesses into manageable ‘challenges’
2.	Identify challenge and define indicators to measure success in addressing that challenge
3.	Choose approach to address challenge
4.	Choose tools to address challenge
5.	Describe and carry out activities and tasks to address challenge
6.	Collect data throughout to monitor and evaluate effectiveness of intervention
7.	Conduct an organizational reassessment and repeat cycle

In the text that follows, partner refers to the local organization (government, non governmental organization, faith based organization, civil society organization, private company, etc), while donor refers to any groups that provide funding to the partner organizations.

### Organizational assessment

The need to conduct organizational assessment prior to engaging in capacity building projects presupposes that any initiative to improve organizational functioning or performance should be in response to an identified organizational need or deficiency [34]. Organizational assessment is the process by which organizations obtain systematic information about their performance and the factors that affect it in order to diagnose competency areas and areas in need of investment [50]. Organizational assessments should be carried out under the partner organization’s leadership in the country-ownership context, to ensure that these organizations can effectively self-assess in the future without the support of external partners.

Organizational assessments can be facilitated by using tools. These tools vary in terms of their focus and approach; some tools assess the organization as a whole while others assess effectiveness one specific area (i.e. governance). Assessments use heterogeneous language to identify and describe the organizational core competencies that are evaluated, similar to the capacity building frameworks previously discussed. Assessments diagnose organizational weaknesses by probing into the organization’s current functioning or performance level and offering indicators that reflect the characteristics of an organization operating efficiently to serve as a comparison.

There are collections of tools available via internet databases, many of which are free for use by the public. There is little information available concerning the validity, quality or effectiveness of each tool, though some tools (like the McKinsey Capacity Assessment Grid) are often cited in the literature and have gained credibility. Existing tools only enable subjective assessments of organizational capacity, which makes objective measurement of capacity which has been built, impossible. Donor and partner organizations should collaboratively identify tools that address areas of relevance to the partner organization and work to ensure the partner organization becomes well versed in conducting organizational assessments.

The goal of the assessment phase is to identify areas of organizational strength and weakness. Once weaknesses have been identified, it is necessary to define the impact that these weaknesses have on organizational performance. Partner organizations should lead the processes of both prioritizing the order in which the identified weaknesses will be addressed and then deconstructing the highest priority weakness into small, manageable ‘challenges’. Capacity building efforts should be designed to address one specific challenge at a time.

### Identify challenge and define indicators to measure success in addressing that challenge

The assessment phase allows organizations to identify a specific challenge(s) on which to focus their capacity building efforts. The challenge will correspond to a target area of intervention, one of the many component parts of the larger core organizational competencies.

Once identified, the organization must design indicators that will demonstrate the effectiveness of the capacity building intervention to stakeholders (boards of directors, funders, beneficiaries etc.). Indicators can vary by type, including process, output, outcome and impact indicators [51]. Indicators provide clear metrics by which the capacity building project can be monitored and evaluated throughout its life. Outcomes are often difficult to link to efforts in practice, as there is rarely a financial bottom line to appraise [22]. Logic models are a commonly used method to identify and define indicators for capacity building. Indicators should be reflective of the specific findings gleaned from the organizational assessment phase.

### Choose approaches and tools to address challenge

A capacity building approach is the general strategy by which an organization is able to address organizational challenges [52]. In the country ownership context, approaches refer to the methods used by the donor/partner organizations collaboratively to help partner organizations better achieve their mission and goals and solve problems that may arise in the future. Capacity building approaches are often boiled down to training and technical assistance provided by the donor organizations; however, there are a myriad of different forms that these approaches can assume. These include professional training, peer assessments, process consulting, performance contracting, executive coaching, mentorship, international organizational collaboration, and a variety of partnership types.

Each approach can be operationalized by means of a variety of tools and activities. For example, a partner/donor organization may decide to address an organizational need for enhanced capacity in governance, which was identified through a process of organizational assessment. Within the arena of governance, this organization might have identified the need to increase and diversify board membership as the challenge to be addressed. Specific indicators would then be defined in order to track the capacity building effort’s progress in addressing that challenge (i.e. the number of different community sectors represented by board members). It is then necessary to identify the desired approach to addressing the challenge of building governance capacity. The chosen approach, (technical assistance around board development for example), is operationalized through use of a specific set of activities and tasks such monthly consultations between the donor and partner organizations regarding sound board member recruitment and retention methods. This tool can be further divided into its component parts, or activities, which could include the preparation of specific questions to pose at the monthly consultation meetings and so on. The application of the defined tools and activities represent the implementation phase of a capacity building project [52,53].

### Evaluate the effectiveness of capacity building efforts

Evaluation in capacity building projects is necessary to increase the accountability of both donor and partner organizations, to allow donor organizations that sponsor capacity building initiatives to engage in more evidence-based grant making, and to compare the effectiveness of different capacity building approaches [22]. Capacity building in the context of country ownership has undergone little formal evaluation and there are numerous explanations for this dearth of evidence. No standardized approaches to monitoring and evaluating capacity building measures are currently defined due to the range of activities and circumstances that comprise capacity building interventions [31]. Monitoring and evaluation is distinct from the initial assessment phase in that it aims to measure change over time whereas the initial assessment is diagnostic and identifies organizational gaps [53]. Difficulties inherent to defining ‘capacity’ and measuring an organization’s progress toward it are major challenges to evaluating effectiveness, particularly when evaluating ‘soft’ capacities, like leadership development or employee motivation, that are challenging to quantify [54-56].

Capacity building projects are often facilitated through grant funding, part of which may or may not be allocated for evaluation purposes by the funder organization [57]. In the absence of designated resources, effective evaluation efforts may be difficult to conduct. Partner and donor organizations may also have disparate understandings of the capacity building initiative’s actual goals and, by extension, how they should be evaluated. The existing evidence base of capacity building evaluations generally describes either success in individual case studies or among small samples of organizations, which hampers the generalizability of their findings [32].

Partner and donor organizations should be prepared to test new strategies and approaches to monitor and evaluate capacity building initiatives [53]. Currently utilized methods range from the traditional donor-driven approaches to the participatory and ultimately self-directed activities of partner organizations; it is the participatory approach that should be embraced in country-owned capacity building [55]. Evaluation activities can serve two integrated purposes; assessing the effectiveness/impact of a specific capacity building intervention and building the partner organization’s capacity to conduct evaluations independently [32]. Because capacity is a multi-faceted construct, it is necessary to remember that any one indicator tells only part of a complex story. To ensure ownership, attempts to capture changes in capacity over time should be pursued through the use of indicators that are defined by the partner organization and relevant to locally determined concepts of change [31].

### Conduct an organizational reassessment and repeat the cycle

Once the organizational challenge has been addressed and evaluated, the organization will have evolved in some manner. Addressing the original identified challenge will ideally result in increased organizational capacity and efficiency; however, capacity building should be continuous process. Improved capacity in one area may expose other areas of organizational weakness that also require intervention, or the other pre-existing weaknesses may still be present. The ‘new’ organization must be re-assessed and another cycle of capacity building activities should commence in order to address the next high-priority organizational challenge identified.

**Figure 1:**
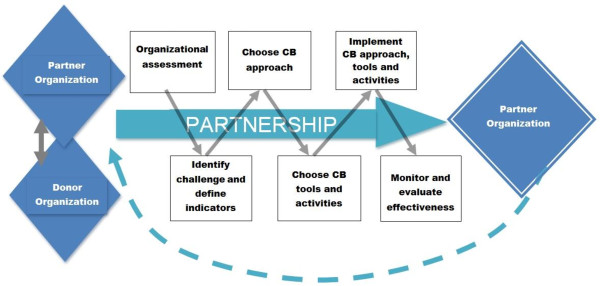
Visual schematic of country-owned capacity building framework.

The above process of partnership between donor organization and local partner should ultimately lead to local organizations capable of conducting the cycle of capacity building activities internally without external guidance or leadership, as described in Figure 1.

### Sustainability

Effective organizational capacity building will contribute to an organization’s sustainability and, in the context of country ownership, enhancing the sustainability of the partner organization and the health outcomes it aims to produce is often a top priority [34]. Capacity is an organization’s ability to perform its defined functions, set and implement developmental objectives, and adapt and respond to changing environments effectively, efficiently and on a sustainable basis [4,16]. Capacity building activities should be designed to endow partner organizations with the skills and abilities to sustain current built capacity, to grow the skills necessary within the organization to continue to build its own capacity, as well as to respond to and proactively create changes in its environment into the future, absent of donor organization and country support [36,55].

Agreement on the importance of building capacity in a sustainable way is necessary from the outset of the partnership between donor and partner organizations [36]. Leadership within the partner organization will ultimately be responsible for directing and stewarding future capacity building efforts; however, buy-in from all levels of the organization and other relevant stakeholders and the identification of internal and external champions of the capacity building process will help ensure its future sustainability [5,23,34]. To ensure that donor organizations impart all relevant expertise, opportunities for technical support and training should be demand-driven and informed by the partner organization’s knowledge and understanding of local context and organizational needs [34]. It should not be assumed that best practices that are effective within the donor organization’s own country or experience will translate seamlessly into the partner organization’s context [21,36].

Training-of-trainer approaches and coaching techniques (that can augment traditional supervision or mentoring relationships by drawing upon the unique knowledge and experiences of both the coach and coached), could also be employed to build in-house expertise among the partner organization’s leadership in any necessary skill set. For example, training several partner organization staff members to train others in skills relating to board development or to pursuing new and strengthening existing partnerships with other organizations will ensure that this technical expertise remains within the organization regardless of staff turnover. Certain competencies, including those concerning organizational finances and strategic management, that contribute directly to an organization’s future sustainability can be prioritized for training and technical support [21]. Donor organizations can assist partner organizations in creating sustainability plans that can quantify the partner organization’s capacity building goals prospectively and provide a framework for measurement and evaluation in achieving those goals, increasing the likelihood of successful and long-lasting organizational change [30].

## Summary

A common theme throughout this paper has been the importance of cultivating partner/donor organization partnership as driving force for country-owned capacity building projects. Partnership presupposes that capacity building can and should be a two-way street, with donor organizations standing to gain knowledge from their relationships with their partners [42]. Participation at all levels of the partner organization will ensure that the capacity building effort is truly owned by the organization; it will build both the organization’s capacity and that of individual employees to implement capacity building processes without donor organization support in the future.

Donor organizations have a responsibility to empower partner organizations to build capacity independently by teaching them to identify areas of needed intervention (through conducting organizational assessments), to plan and implement a capacity building process (through identifying a specific organizational challenge, setting indicators, deciding on data collection methods, and choosing approaches, tools and activities) and to evaluate the effectiveness of their efforts. Donor organizations should anticipate any cultural considerations that could hinder partnership efforts. Donor organizations may need to simplify or reorganize their own internal processes to facilitate collaboration. Aligning to the partner organization’s priorities, systems and procedures is itself a capacity that donor organizations must develop [36].

Partner organizations are expected to take the lead in country-owned capacity building. In this sense, leadership is an organizational capacity of primary importance, as future capacity building efforts cannot occur without effective leadership. Leadership development, like monitoring and evaluation, should be built into every capacity building intervention as an integral part of the process and also as a desired outcome. Similarly, financial management skills are another capacity that should receive due attention in capacity building partnerships, particularly in light of the GHI funding that partner organizations will ultimately be accountable for managing.

Country-owned capacity building is a relatively new concept that requires further theoretical exploration. The Paris Declaration on Aid Effectiveness is just that – a declaration. It details the principles of country ownership to which partner and donor countries should commit, but not the specific mechanisms to carry out these principles. More evidence as to how country-owned capacity building plays out in practice is needed to guide future interventions. Donor organizations must build funding for evaluative purposes into their grants and the results of effective interventions should not die unseen in internal reports. Evidence documenting successes and challenges should be made available to other organizations as guide posts to prevent the duplication of ineffective past efforts. It is the organizational actors on the ground that are poised to identify, evaluate and disseminate the best practices that emerge in the practice of country-owned capacity building. The Global Health Initiative funding that is currently underway is an opportunity to collect evaluative data and establish a centralized and comprehensive evidence base that could be made available to guide future country-owned capacity building efforts.

## Abbreviations

GHI, Global Health Initiative; CB, Capacity Building; OD, Organizational Development.

## Competing interests

Both Authors declare that they have no competing interest.

## Authors contributions

MB conceived the original study, provided guidance and comments as the paper was developed, and contributed to the final text. JG conducted the literature review, interviewed key informants, synthesized the literature into the concepts expressed in the paper and was the primary author. All authors read and approved the final manuscript.

## Pre-publication history

The pre-publication history for this paper can be accessed here:

http://www.biomedcentral.com/1471-2458/12/531/prepub

## References

[B1] U.S. Department of State Leading Through Civilian Power: The First Quadrennial Diplomacy and Development Review 2010 Department of State, Washington (DC); United States [cited 2012 July 17] Available from http://www.state.gov/documents/organization/153108.pdf

[B2] United States Agency for International Development Implementation of the global health initiative: consultation document 2012 United States Department of State, Washington (DC) [cited 2012 July 17] Available from http://www.pepfar.gov/documents/organization/136504.pdf

[B3] Cohen JM Building Sustainable Public Sector Managerial, Professional, and Technical Capacity: A Framework for Analysis and Intervention 1993 473 Harvard Institute for International Development; 1993. Development Discussion Papers, Harvard University

[B4] Philbin A Capacity Building in Social Justice Organizations Capacity Building in Social Justice Organizations, by Ann Philbin 1996 Ford Foundation, New York, New York

[B5] Light P Hubbard E The Capacity-Building Challenge Part I: A Research Perspective 2004 Foundation Center, New York

[B6] McPhee P Bare J DeVita CJ, Fleming C Introduction Capacity Building in Nonprofit Organizations 2001 The Urban Institute, 1 3 [Cited 2012 July 17] Available from [http://www.urban.org/uploadedpdf/building_capacity.pdf]

[B7] Fine A Kopf N Thayer C Echoes from the Field: Proven Capacity-Building Principles for Nonprofits 2001 Environmental Support Center, Washington, DC http://www.impactalliance.org/ev02.php?ID=19879_201&ID2=DO_TOPIC

[B8] LaFond A Brown L A Guide to Monitoring and Evaluation of Capacity-Building Interventions in the Health Sector in Developing Countries. MEASURE Evaluation Manual Series 2003 No. Carolina Population Center, University of North Carolina at Chapel Hill [Cited 2012 July 17] Available at http://www.cpc.unc.edu/measure/publications/ms-03-07

[B9] Connolly P Lukas C Strengthening Nonprofit Performance: A funder’s Guide to Capacity Building 2002 Amherst Wilder Foundation, St. Paul, MN

[B10] Hudson M Managing at the Leading Edge: new Challenges in Managing Nonprofit Organizations 2005 Jossey-Bass, San Francisco, CA

[B11] Campobasso L Davis D Reflections on Capacity Building 2001 The California Wellness Foundation, Woodland Hills, CA

[B12] United Nations Development Program Capacity Assessment and Development in a Systems or Strategic Management Context: Technical Advisory Paper No.3 Management Development and Governance Division Bureau for Development Policy 1998 UNDP, New York

[B13] Backer TE DeVita CJ, Fleming C Strengthening nonprofits: foundation initiatives for nonprofit organizations Building Capacity in Nonprofit Organizations 2001 Urban Institute, Washington, DC

[B14] Hildebrand ME Grindle MS Building Capacity: Challenges for the Public Sector 1994 Harvard University, Harvard Institute for International Development

[B15] Linnell D Evaluation of Capacity Building: Lessons from the Field 2003 Alliance for Nonprofit Management, Washington, DC

[B16] Milen A What do we Know About Capacity Building? An Overview of Existing Knowledge and Good Practice 2001 Department of Health Service Provision, World Health Organization, Geneva http://www.unescobkk.org/fileadmin/user_upload/aims/capacity_building.pdf

[B17] Boffin N Health System Capacity Building: Review of the Literature DGOS—AIDS IMPULS PROGRAM 97203 BVO 2002 Institute of Tropical Medicine, Antwerp, Belgium http://www.itg.be/itg/uploads/publichealth/Review%20health%20system%20capacity%20building.pdf

[B18] Alfirevic N Gabelica N Management practices in Croatian non-profit organizations: results of the empirical research J ContempManag Issues 2007 12 1 25

[B19] Sunje A Pasic M Modeling an Organizational Sustainable Competitive Advantage. An Enterprise Odyssey.International Conference Proceedings.Zagreb 2004 1535 Faculty of Economics and Business, University of Zagreb

[B20] Fuller R Russ-Eft D Organizational responsiveness of Russian and American growth-oriented small and medium enterprises (SMEs) Hum Resour Dev Int 2010 13 3 317 10.1080/13678868.2010.483820

[B21] Hulme D Enhancing organizational effectiveness in developing countries: the training and visit system revisited Public Adm Dev 1992 12 5 433 445 10.1002/pad.4230120503

[B22] Connolly P York P Building the capacity of capacity builders: a study of management support and field-building organizations in the nonprofit sector 2003 The Conservation Company, New York [cited 2012 July 17] Available at http://www.tccgrp.com/pdfs/buildingthecapacityofcapacitybuilders.pdf

[B23] Heward S Hutchins C Keleher H Organizational change – key to capacity building and effective health promotion Heal PromotInt 2007 22 2 170 178 10.1093/heapro/dam011 17495992

[B24] York PJ Learning as We Go: Making Evaluation Work for Everyone 2003 TCCgroup Briefing Paper, Philadelphia [cited 2012 July 17] Available at http://www.tccgrp.com/pdfs/per_brief_lawg.pdf

[B25] World Health Organization The World Health Report 2000. Health Systems: Improving Performance 2000 World Health Organization, Geneva [cited 2012 July 17] Available at http://www.who.int/whr/2000/en/whr00_en.pdf

[B26] Wetta-Hall R Ablah E Oler-Manske J Berry M Molgaard C Strategies for community-based organization capacity building: planning on a shoestring budget Health Care Manag 2004 23 4 302 309 10.1097/00126450-200410000-00003 15638337

[B27] Holcombe SH Nawaz SA Kamwendo A Ba K Managing development: NGO perspectives? Int Public Manag J 2004 7 2 187

[B28] Yiannis ES Gregory P Understanding organizational capabilities: towards a conceptual framework Journal of Knowledge Management 2004 8 3 31 43 10.1108/13673270410541024

[B29] Cashman RA Durant JF Theorizing limits: an exploration of boundaries, learning, and emancipation J Organ Chang Manag 2003 16 6 650 10.1108/09534810310502586

[B30] Moran JW Brightman BK Leading organizational change.Career Dev Int 2001 6 2 111 119

[B31] LaFond AK Brown L Macintyre KK Mapping capacity in the health sector: a conceptual framework Int J Heal Plan Manag 2002 17 3 22 10.1002/hpm.649 11963442

[B32] Schermerhorn JR McCarthy A Enhancing performance capacity in the workplace: a reflection on the significance of the individual Ir J Manag 2004 25 2 45

[B33] DeVita C Fleming C Twombly E Building Capacity in Nonprofit Organizations 2001 The Urban Institute Press, Washington, DC

[B34] AIDSTAR-Two Organizational Capacity Building Framework: A Foundation for Stronger, More Sustainable HIV/AIDS Programs Organizations & Networks 2011 Management Sciences for Health, Washington (DC) http://www.aidstar-two.org/upload/AS2_TechnicalBrief-2_4-Jan-2011.pdf

[B35] Light PC Sustaining Nonprofit Performance: The Case for Capacity Building and the Evidence to Support it 2004 Brookings Institution Press, Washington, DC

[B36] Organization of Economic Cooperation and Development The Paris Declaration for aid Effectiveness and the Accra Agenda for Action 2008 Organization for Economic Co-operation and Development, Paris [cited 2012 July 27] Available at http://www.oecd.org/document/18/0,3746,en_2649_3236398_35401554_1_1_1_1,00.html

[B37] Booth D Country Ownership When There is no Social Contract: Towards a Realistic Perspective 2010 Paper presented at the SID-Netherlands Lecture Series 'Global Values in a Changing World, Amsterdam

[B38] Johnson OEG Country Ownership of Reform Programs and the Implications for Conditionality. Research Paper 2005 G-24 Intergovernmental Group of 24, Washington, D.C

[B39] Buiter WH Country ownership: a term who’s time has gone Dev Pract 2007 17 4/5 647 652

[B40] Atun R Kazatchkine M Promoting country ownership and stewardship of health programs: the Global Fund experience J Acquir Immune DeficSyndr 2009 52 Supp 1 S67 S68 10.1097/QAI.0b013e3181bbcd58 19858945

[B41] Obsanyo JO Leadership and Management – Capacity Building and Country Ownership 2011 Health Systems 20/20 Project, Washington (DC) [cited 2012 July 17] Available at http://www.healthsystems2020.org/content/resource/?type=51

[B42] Sanyal P Capacity building through partnership: Intermediary nongovernmental organizations as local and global actors Nonprofit Volunt Sect Q 2006 35 1 66 82 10.1177/0899764005282480

[B43] Health Systems 20/20 Conceptual framework for institutional capacity building 2011 Health Systems 20/20 Project, Washington (DC) [cited 2012 July 17] Available at http://www.healthsystems2020.org/section/topics/capacity/framework

[B44] Nu’Man J King W Bhalakia A Criss S A framework for building organizational capacity integrating planning, monitoring, and evaluation J Public Health ManagPract 2007 S24 32 10.1097/00124784-200701001-00006 17159464

[B45] Lusthaus C Institutional Assessment: A Framework for Strengthening Organizational Capacity for IDRC's Research Partners 1995 IDRC, Ottawa

[B46] McKinsey & Company for Venture Philanthropy Partners Effective Capacity Building in Nonprofit Organizations 2011 Venture Philanthropy Partners, Washington (DC) [cited 2012 July 17] Available at http://vppartners.org/learning/reports/capacity/full_rpt.pdf/

[B47] Sobeck J Agius E Organizational capacity building: addressing a research and practice gap EvalProg Plan 2007 30 237 246 10.1016/j.evalprogplan.2007.04.003 17689329

[B48] Create the Future Web Site Capacity Building Framework 2011 [Internet] [cited 2012 July 17] Available at http://www.createthefuture.com/Capacity_Building.htm

[B49] UNESCO Capacity Building Framework 2006 [Internet] U N E S C O -International Institute for Capacity Building in Africa, Addis Ababa [cited 2012 July 17] Available at http://unesdoc.unesco.org/images/0015/001511/151179eo.pdf

[B50] Morgan P Tascherwau S Capacity and Institutional Assessment: Frameworks, Methods and Tools for Analysis 1996 Canadian International Development Agency, Policy Branch, Ottawa [cited 2012 July 17] Available at http://citeseerx.ist.psu.edu/viewdoc/summary?10.1.1.119.7536

[B51] Ritchey-Vance M Blauert J, Zadek S, Blauert J, Zadek S Widening the lens on impact assessment: the inter-American foundation and its grass roots development framework – the cone. (Chapter 3) Mediating Sustainability: Growing Policy from the Grass Roots 1998 Kumarian Press, Sterling, Virginia

[B52] Eade D Capacity-Building: An Approach to People Centered Development 2005 Oxfam UK and Ireland, UK and Ireland 30 39

[B53] Honadle BW A capacity-building framework: a search for concept and purpose Public Adm Rev 1981 41 5 575 580 10.2307/976270

[B54] Wing KT Assessing the effectiveness of capacity-building initiatives: seven issues for the field Nonprofit Volunt Sect Q 2004 33 153 160 10.1177/0899764003261518

[B55] Morgan P An Update on the Performance Monitoring of Capacity Development Programs: What are we Learning? Paper Presented at the Meeting of the DAC Informal Network on Institutional and Capacity Development 1999 CIDA Policy Branch, Ottawa

[B56] Gariba Jackson ET, Kassam Y Participatory impact assessment as a tool for change: lessons from poverty alleviation projects in Africa. In Knowledge Shared Participatory Evaluation in Development Cooperation 1998 IDRC, Ottawa 64 81 [cited 2012 July 17] Available at http://web.idrc.ca/openebooks/868-6/

[B57] Kibbe BD Enright KP Lee JE Culwell AC Sonsini LS Speirn SK Tuan MT Funding Effectiveness: Lessons in Building Nonprofit Capacity, by Grantmakers for Effective Organizations 2004 Jossey-Bass, San Francisco, CA

